# A new kink in an old theory of carcinogenesis

**DOI:** 10.1186/1742-4682-10-12

**Published:** 2013-02-18

**Authors:** Richmond T Prehn, Liisa M Prehn

**Affiliations:** 1 Department of Pathology, University of Washington, 5433 S. Hudson St, Seattle, WA, USA

## Abstract

According to Berenblum’s two-stage hypothesis, the first stage in carcinogenesis is the production of benign premalignant lesions. Between this initiation stage and the formation of a malignant tumor there is often a long lag phase. We propose that this lag is caused by the delay in the formation of a new and rare tumor-specific antigen, which induces an immune response that *stimulates* tumor growth. Such tumor-specific antigens could arise as a result of a mutator-like phenotype, which is supposedly present in the benign initial stage of carcinogenesis. According to this hypothesis, the first stage lesion provides a weakly mutagenic environment conducive to the formation of the new antigen(s). If no such new antigens appear so there is no consequent immune response, it is argued that carcinogenesis would seldom if ever ensue.

## Background

The phenomenon upon which this discussion is based is nicely summarized in a paper by Brash and Cairns [1], a short section of which is reproduced here: “… Humans and animals show the same strange relationship between dose of carcinogen and time of appearance of their cancers. For example, although the incidence of lung cancer in smokers appears to be directly proportional to the number of cigarettes smoked per day [2], it is proportional to roughly the sixth power of the duration of smoking. Similarly, when rats are continuously exposed to dietary carcinogens their incidence of cancer rises as the first or second power of dose rate but as a much higher power of time [3,4] … smokers keep their raised rate of lung cancer for many years after they have stopped smoking [5] … These numerous experiments suggest, therefore, that mutagenic carcinogens cause just one or two events and these (like the initial event in in vitro carcinogenesis) are then followed by steps that accumulate solely with the passage of time, driven perhaps by cell division [6]…”.

In the present paper we review the probability that the frequently observed time lag in carcinogenesis is caused by the delay in the formation of a new tumor-specific antigen that, in turn, induces a tumor-stimulating immune response [7]. This tumor-specific antigen is postulated to arise as a result of a mutator-like phenotype that we postulate exists in the benign first stage of the carcinogenic mechanism [7,8].

In 1949–50, when one of the present authors (RTP) joined the laboratory of the late Dr. H.B. Andervont as a fellow at the National Cancer Institute, interest was very low in all aspects of immunity in relation to cancer. In fact, in about 1956, RTP was told by the head of the National Cancer Institute that “you would be well advised to find some other topic for your investigations, many a career has been dashed on those hard rocks” - a vote of no confidence that led directly to RTP leaving the Institute. However, it was probably sound and proper advice at that time. After the paper of Prehn and Main in 1957 [9], followed shortly by that of the Karolinska group showing that tumor-specific immunity was possible against a methylcholanthracine-induced sarcoma even in the autologous mouse [10], the immune surveillance hypothesis became popular and has remained so to the present day.

### Role of the mutator phenotype in carcinogenesis

The recent discovery of thousands of mutations in each cancer has suggested one plausible explanation for the often long delay in carcinogenesis; an ongoing mutagenic environment was probably created during an earlier exposure to mutagens (in all likelihood by the failure, via mutation, of a gene repair mechanism [8]). The resulting mutator phenotype would eventually and inevitably result in a combination of mutants that must grow as a cancer. This hypothesis accounts nicely for the lag so often seen in carcinogenesis. Other mechanisms to enhance carcinogenic accumulations have also been proposed [11-13], but the question remains - is there some further plausible explanation for the sometimes long delay beyond the time required for the mere accumulation of a carcinoma genome? Present data suggest that such an explanation may reside within the immune system.

### Role of the immune system in oncogenesis

A further hypothesis that might corroborate the mutator phenotype hypothesis is a gradual decline in the strength of the immune mechanisms with age; a decline in immunity would eventually result in a cluster of mutations that would be able to grow in that weakened immune environment. Under this hypothesis, the role of time can be accounted for by the known decline in immune mechanisms in adults with increasing age [14]. It also is apparent that the incidence of most cancers rises with increasing age; it is obvious that these facts could be causally related.

We have previously published evidence suggesting that the primary role of the immune reaction in carcinogenesis is to stimulate rather than to inhibit cancer growth [15]. This stimulatory role for immunity in no way negates the idea that a decline in the immune capacity of the host may cause the eventual appearance of a cancer. Immunostimulation is dependent upon the quantitative ratio of immune reactants to tumor size; in general - up to a point - the weaker the immune mechanism, the greater the stimulatory effect on tumor growth. Thus, the weaker immunity in the aged might well be stimulatory to incipient cancers [16].

Until recently, it seemed very unlikely that immune mechanisms could affect human tumorigenesis because immune effects seemed largely confined to relatively rare carcinogen- or virus-induced cancers [17]. Hewitt was vociferous on the subject; he went so far as to publish his failed attempt to demonstrate the induction of immune inhibition in seven different spontaneous cancers in mice; the only effect of his immunization attempts was that, in all seven cases that he tested, the tumors grew better in the presumptively immunized syngeneic mice than in the non-immunized controls [17]! This same stimulation of the growths of implants in specifically immunized recipients was confirmed using a new set of spontaneous mouse tumors by workers in Argentina [18]. Hewitt suggested that his own results were due to a lack of specific antigens on his spontaneously-derived tumors. However, we interpreted the stimulation as showing the presence of low levels of tumor-specific antigens that produced weakly stimulatory rather than inhibitory concentrations of immune reactants. As already mentioned, in 1972 RTP had made the counterintuitive observation that small numbers of spleen cells from tumor immune mice could, when mixed with the target tumor, sped rather than inhibited the growth of the mixture when it was implanted into immunodepressed syngeneic recipients [15]. Apparently the immune system is subject to some form of hormesis because larger quantities of immune cells, when used in like manner, markedly inhibited the cancer’s growth [19]. This observation has, in essence, been confirmed by many others [20-22] and is central to the present formulation.

### The two-stage hypothesis

In the 1950s, a major topic of discussion was the idea put forth by Friedewald and Rous [23], and further developed by Berenblum and Shubic [24], that carcinogenesis could be divided into two quite different and distinct stages. There has been much subsequent argument about the phenomenon, but it is widely agreed that it is real, at least in many situations. The essence of the proposal is that if a sub-carcinogenic dosage of a carcinogen is followed by one of a number of non-carcinogenic irritants, cancers may then be produced; classically, a subcarcinogenic dosage of a hydrocarbon carcinogen (the initiator), followed by paintings of the same skin area with the non-carcinogenic irritant, croton oil (the promoter). The initiating effect was shown to be very long lasting. However, reversal of the application sequence yielded no observable carcinogenic effect in most studies. Students in our laboratory devised another method that used skin grafting as a promoter [25,26].

According to Berenblum’s two-stage hypothesis [24], the first stage is the production of benign premalignant lesions. We postulate that the second stage is a small immune response to rare new antigens on a previously benign first stage (papilloma?) cell, which stimulates that cell to multiply as a malignant tumor. According to this hypothesis, the papilloma or other benign first stage lesion would itself provide an environment conducive to the formation of new antigens (a weakly mutagenic environment); in the absence of such new antigens and the consequent immune response to them, carcinogenesis would, according to the work of Andrews, seldom if ever occur [7,26]. Andrews’s two stages produced no cancers but many regressing benign papillomas in mice that had been *maximally* immunodepressed [27]. To achieve an immunological cancer cure, one would apparently have to supply the patient with a *large enough* immune reaction to inhibit rather than stimulate tumor growth. Alternatively, perhaps one could inhibit tumor growth by *drastically* reducing the immune reaction.

## Conclusions

Cancer formation probably depends upon a new antigenicity that arouses a weak but stimulatory immunity to a new tumor antigen - an antigen that arose as a kink in the benign cells that had been induced in the first stage of the two stage carcinogenic process [7]. This kinked two stage hypothesis accounts rather neatly for the time lag in the carcinogenic mechanism.

The reality of the phenomenon of immunostimulation of cancer by relatively small quantities of immunity is now well established [20-22], though the mechanism of this stimulatory effect remains obscure. A possible explanation may reside in work of Rubin showing that cellular growth is inhibited by interactions with surrounding normal cells [28]; perhaps a small amount of immunity can interfere with this inhibitory reaction and thus appear to stimulate [29].

Possible mechanisms of immunostimulation have also been discussed by Parmiani [30]. We acknowledge that immunity is a very complex phenomenon; many diferent types of immune cells have been described and, although in this paper we have emphasized the overriding importance of the quantitative relationship between the target and immunity, we admit that among the complexities of the response are individual elements that may be stimulatory while others are inhibitory to tumor growth [31-33]. It seems possible to us that whether an immune effector becomes a morphologically recognizable inhibitor or stimulator might even be determined, at least in part, by the afore mentioned quantitatve relationship, albeit that we have little evidence for such an effect.

It seems probable that selection might, in most cases, keep a weak immune reaction at nearly optimal levels for tumor growth indefinitely. However, the fact that a depressed level of immunity increases the growth of some tumor types in humans [34,35] suggests that such tumors may not have originally enjoyed the optimal immunity for the fastest possible tumor growth.

## Competing interests

There are no competing interests.

## Authors’ contribution

Both authors were equally responsible for all parts of the M.S. Both authors read and approved the final manuscript.
